# Finding the Link between Cyberbullying and Suicidal Behaviour among Adolescents in Peninsular Malaysia

**DOI:** 10.3390/healthcare10050856

**Published:** 2022-05-06

**Authors:** Siti Aisyah Mohd Fadhli, Jasy Liew Suet Yan, Ahmad Shahril Ab Halim, Asrenee Ab Razak, Azriani Ab Rahman

**Affiliations:** 1 Department of Community Medicine, School of Medical Sciences, Universiti Sains Malaysia, Kubang Kerian 16150, Kelantan, Malaysia; aisyah021096@gmail.com; 2 School of Computer Sciences, Universiti Sains Malaysia, Gelugor 11800, Penang, Malaysia; jasyliew@usm.my; 3 Department of Psychiatry, School of Medical Sciences, Universiti Sains Malaysia, Kubang Kerian 16150, Kelantan, Malaysia; shahrilabhalim@usm.my (A.S.A.H.); asrenee@usm.my (A.A.R.)

**Keywords:** cyberbullying, suicidal behaviour, adolescents, Peninsular Malaysia, COVID-19 pandemic

## Abstract

Social media engagement has contributed to the rise of cyberbullying, which has recently triggered tragic suicides among adolescents. The objective of this cross-sectional study is to determine the prevalence of cyberbullying, suicidal behaviour, and their association among adolescents in Peninsular Malaysia. The study was conducted among 1290 secondary school adolescents aged between 13 and 17 years old in Peninsular Malaysia using a self-administered and anonymous online questionnaire. Our findings reveal that the prevalence of cyberbullying victimization and perpetrator is 13.7% and 3.8%, respectively. The prevalence of suicidal behaviour among adolescents is 17.1%, in which 11.9% had suicidal thoughts, 10.2% had a suicide plan, and 8.4% had made a suicide attempt. Multiple logistic regression shows that adolescents experiencing cyberbullying victimization is a significant risk factor (*p* < 0.001) for suicidal behaviour after adjusting for other confounders. An alarming number of adolescents in Peninsular Malaysia found to be involved in cyberbullying and its significant association with suicidal behaviour warrant the need to strengthen current interventions. Since the study was conducted during the COVID-19 pandemic, our findings make an important contribution in reporting current trends in cyberbullying and suicide among adolescents, especially when they are becoming ever-more present in cyberspaces.

## 1. Introduction

Cyberbullying is a new way for young people to express their dissatisfaction, often in a violent manner, as it is becoming easier to gain access to information and communication technologies. It is defined as an offensive, premeditated act performed on a victim who is unable to defend themselves via electronic communication technology on a regular and ongoing basis by an organisation or individual [1]. Globally, the common occurrence of cyber-victimization ranges from 5% to 59% [2,3], and cyber-offenders range from 6% to 46% [3]. A survey conducted by UNICEF on cyberbullying involving more than 170,000 adolescents and young adults in 30 countries in 2019 reported that one third of youth were identified as victims of cyberbullying [4].

Several studies from different countries reported that cyberbullying was found to have a negative impact on mental health such as depression [5,6,7,8], which might lead to suicidal behaviour in both the aggressors and victims [9]. The link between cyberbullying and suicidal behaviour can be explained using the interpersonal theory of suicide [10], which proposed that individuals’ suicidal ideation or desire for suicide heightened when experiencing uncontainable feelings of two potent interpersonal suicide risk factors, thwarted belongingness, and perceived burdensomeness. The presence of suicidal desire and the capability for it then leads to lethal or near-lethal suicidal acts [10]. Thwarted belongingness is a sense of loneliness and the absence of a reciprocally caring relationship, whereas perceived burdensomeness refers to the belief that the self is so flawed as to be a liability to others, and affectively laden cognitions of self-hatred. Two studies examined the implications of cyberbullying victimization and suicidal behaviour with depression as the mediator using the interpersonal theory of suicide [11,12], in which cyberbullying victimization was identified as profound interpersonal stress that could lead to adolescent loneliness, social disengagement [13], and low self-esteem [14], all of which are key components in the interpersonal theory of suicide [11,12]. As a result, the increased degree of thwarted belongingness and perception of burdensomeness are linked to an increased risk of suicide among teenagers who have been cyber-victimized when mediated by depression [11,12]. We have not yet found any studies applying the theory to explain the link between cyberbullying perpetrators and suicide.

Even though the studies exploring cyberbullying and suicidal behaviour using the interpersonal theory of suicide are limited, a number of studies proved that cyberbullying perpetrators or victims were strongly associated with suicidal behaviour among adolescents [8,9,15,16]. Cyberbullying victims or perpetrators had double the risk of suicidal behaviour than those not involved in cyberbullying [15]. Despite the positive association shown between cyberbullying and suicidal behaviour, cyberbullying does not directly cause suicide [17]. Cyberbullying was associated with psychiatric illnesses such as depression [5,6,7,8], anxiety [18], and stress [19], which may predispose to suicidal behaviour among adolescents [9].

Suicide occurs all over the globe in low-to-high-income countries. Approximately more than 70% of suicide incidences occurred in low- and middle-income countries [20]. Suicide is the fourth leading cause of death among individuals aged 15 to 29 [21] and third leading cause of death among female adolescents aged 15 to 19 years old [21], making it a global public health concern. Suicidal behaviour among adolescents is influenced by multiple factors including individual factors (age, gender, academic pressure, social media, depression), family factors (family environment, parental marital status, household income, history of abuse), cyberbullying and socio-environmental factors (type of school, social support from parents and peers, school locality) [22,23]. Prior studies reported that apart from bullying, suicidal behaviour among adolescents has also been linked to other factors including having perceived academic pressure [23], no close friends [24], gender [25,26,27], poor household income [27,28,29], urban households [30], history of abuse [31,32,33], lack of parental support [34,35], lack of peer support [36,37], history of substance abuse [25,29,33] and unstable family environment [38,39,40]. These studies reported that adolescents who had an unstable family environment [38,39,40], poor household income [27,28,29], history of abuse [31,32,33], and lack of social support from family [34,35] and friends [36,37] had higher odds of suicidal behaviour. Adolescents who had depression were four times more at risk of suicidal behaviour than those who did not have depression [34]. Several research studies on identifying risk factors of suicidal behaviour among adolescents reported that perceived academic pressure [23], being female [25,26] and having a social media account [41] were positively associated with suicidal behaviour among adolescents.

To our knowledge, no existing study has yet to observe any association between the type of school and suicidal behaviour among adolescents. However, suicidal behaviour is positively associated with stress among adolescents [33]. This can be linked to the type and level of stress experienced by adolescents attending different types of school. A local study on stress among secondary school adolescents in Kelantan, Malaysia [42], reported that students who attended religious schools were exposed to a higher level of academic-related stressors, which caused moderate-to-high stress compared with other schools. This was followed by boarding schools and then national schools as the students put high importance on achieving excellent academic performance. Hence, academic stressors may trigger suicidal behaviour [43]. In addition, a local national survey on adolescent health reported that adolescents who lived in urban areas had a higher incidence of suicidal behaviour compared to those who lived in rural areas [30].

The Malaysia National Cybersafe Schools Survey 2013 found that a quarter of school children have frequently encountered online bullying through Facebook, blogs and instant messaging [44]. Recent statistics in Malaysia have shown that 25% of adolescents in Malaysia have been scarred as victims by moderate-to-serious online bullying, whereas as many as 54% have expressed a propensity to be a cyberbully [45]. The COVID-19 pandemic has been connected to mental health difficulties as the consequences of physical distance and stay-at-home orders. According to the 2019 National Health and Morbidity Survey (NHMS), 424,000 children had mental health issues during the pandemic [46]. The Ministry of Health reported 465 attempted suicide cases between January and June 2020, but statistics from The Befrienders Kuala Lumpur revealed more individuals feeling distress and suicidal in July, August, and September 2020 than the previous three months [47]. Living in the new norm in which most school activities, particularly learning, teaching, and socializing, are conducted entirely online has resulted in adolescents becoming more vulnerable to cyberbullying as they are spending more time online [8], thus increasing the risk of suicidal behaviour. Three out of every ten young people in Malaysia are victims of cyberbullying, which occurs most frequently on social media platforms [4]. According to these statistics, cyberbullying has become a substantial threat to the well-being of Malaysian adolescents, and suitable actions are required to prevent additional harm from online aggression.

Research on cyberbullying is relatively new; hence, much is still unknown in Asian countries including Malaysia regarding the consequences of cyberbullying [48,49] on mental health, particularly its link to suicidal behaviour. Thus, the aim of this study is to examine the prevalence of cyberbullying and suicidal behaviour as well as the association between the two phenomena among adolescents in Peninsular Malaysia.

## 2. Materials and Methods

### 2.1. Study Design and Participants

**Figure 1:**
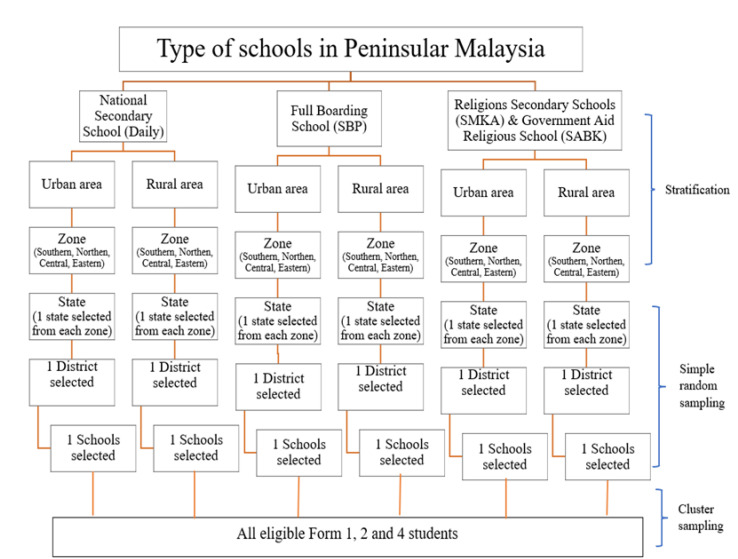
Sampling method and subject recruitment.

A cross-sectional study was conducted in 11 states involving 17 districts and 24 government secondary schools in Peninsular Malaysia. The subjects were selected via multi-stage stratified cluster sampling. Stratification was performed according to types of school (national daily secondary schools, full boarding schools, and religion secondary schools), localities (urban and rural) and zones (eastern, northern, southern, and central). Simple random sampling was used to select one state from each zone and one district from each state within each stratum. From each district, one school was selected by simple random sampling. In each selected school, all eligible students were included as shown in Figure 1. The inclusion criteria encompassed students aged between 13 and 17 years old who were Malaysian citizens, literate, and must be able to understand the Malay language (i.e., the national language). Those with any conditions that prevented understanding or completion of the questionnaires such as severe intellectual disability, pervasive developmental disorder and uncontrolled hyperactivity or inattention were excluded from the study.

### 2.2. Sample Size Calculation

The sample size was determined using the single proportion formula [50] using the prevalence of 52.2% based on an estimated prevalence of cyberbullying [51], a design effect of two to allow for a clustering effect, a precision of 0.04 and a drop-out rate of 20%. Based on this sample size calculation, a total of 1440 respondents were required for the study.

### 2.3. Questionnaire and Measures 

The questionnaire for data collection included a study proforma, the Malay version of the Cyberbullying Scale (CBS-M) and the Malay version of Patient Health Questionnaire-9 (PHQ-9). 

#### 2.3.1. Suicidal Behaviour

The socio-demographic profile and suicidal behaviour of the respondents were acquired using a study proforma. Suicidal behaviour was assessed by asking the following questions (NHMS, 2017 [52]): 

(1) Suicidal ideation was examined by asking “Have you ever seriously considered trying to commit suicide in the past 12 months?”. 

(2) Suicide planning was examined by asking “Have you ever made a plan in the past 12 months about how you would attempt suicide?”. 

(3) Suicide attempt was examined by asking “In the last 12 months, have you ever tried committing suicide?”.

Suicidal behaviour was the dependent/outcome variable in this study with the binary outcome of ‘yes’ and ‘no’. Variables assessing suicidal ideation and plan had a binary “yes” or “no” response. Suicidal ideation was defined as any “yes” response to the question of “Have you ever seriously considered trying to commit suicide in the past 12 months?”. Suicide plan was defined as any “yes” response to the question of “Have you ever made a plan in the past 12 months about how you would attempt suicide?”. For suicide attempt, the answer was divided into five options based on frequency: (1) none, (2) once, (3) two-three times, (4) four-five times, and (5) six and above. For suicide attempt, those who answered ‘none’ was considered as ‘no’ response and those who answered once and above were considered as a ‘yes’ response. 

Therefore, suicidal behaviour was defined as a “yes” response to any suicidal behaviour item, either ideation or plan or attempt. The responses were categorized as yes (1) or no (0). 

#### 2.3.2. Cyberbullying Victimization and Perpetrators

Cyberbullying was assessed using CBS-M, which consisted of two general questions and fourteen items measured with five Likert-type scales ranging from ‘0 = never’ to ‘4 = all the time’. The first two questions were objective-based questions asking for the medium used by students to cyberbully others and where individuals were cyberbullied by others. Summing the individual raw scores for items 3 through 16 yielded a total score with higher values indicating more frequent experiences of being a cyberbullying victim [53,54]. CBS-M was validated by [55] among secondary school adolescents in one of the districts in Malaysia. It had good psychometric properties and good internal consistency (Cronbach’s alpha: 0.87, construct reliability: 0.832).

Cyberbullying victimization and perpetrators were independent variables of interest in this study. Cyberbullying victimization was defined as those who responded to any item in “Have other children used any of the following items to bully you?’ in CBS-M. Cyberbullying perpetrators were defined as those who responded to any item in “Have you used any of the following items to bully other children?’ in CBS-M. Respondents who responded to any items in both cyberbullying victimization and perpetrators were classified as cyberbullying victimization perpetrators. The frequency of cyberbullying victimization was categorized into two categories according to the median score of the CBS-M. Respondents who scored ≤ 2 were classified as less frequent and respondents who scored > 2 were classified as more frequent victims of cyberbullying. 

#### 2.3.3. Depression

Depression was assessed using the Malay version of PHQ-9. It contained self-report measurements for nine questions based on the nine DSM-IV criteria for the diagnosis of major depression. The nine items in PHQ-9 focused on symptoms experienced during the two weeks prior to answering the questionnaire. Each item came with four answer options (“0: not at all”, “1: several days”, “2: more than half the days”, and “3: nearly every day”), which sums up to a total score between the range of 0 to 27. Respondents with scores of 10 and above were categorized as having depression [56,57]. The Malay version of PHQ-9 used to determine depression in this study was validated with good internal reliability (Cronbach’s alpha of 0.70). The Malay version of PHQ-9 had a sensitivity of 87% and a specificity of 82% (74% to 88%) [56]. Respondents with scores of 10 and above were categorized as having depression [56,57].

#### 2.3.4. Sociodemographic, Depression Status and Other Factors Associated with Suicidal Behaviour (Potential Confounders)

Other independent variables considered to be potential confounders included the parental marital status, household income, school locality, type of school, age, gender social support from family members, perceived social support from friends, history of abuse (physical/mental/sexual), perceived academic pressure, social media account ownership, parents fighting in front of adolescents, and depression.

### 2.4. Data Collection

The data were collected from May until September 2021 using a self-administered anonymous online questionnaire. The schools were temporarily closed from all educational activities during the data-collection period because of the movement control order (MCO) due to the COVID-19 pandemic. After obtaining consent from parents and students, the online questionnaire was distributed to the selected students through the school counsellors, who were given instructions by the primary researcher to explain the details of the research to both the parents and students. Any student’s inquiry regarding the questionnaire was forwarded to the primary researcher for clarification through the respective school counsellors. 

### 2.5. Statistical Analysis

Data entry and analysis used IBM SPSS Statistic for Windows, Version 26 (IBM Corp. in Armonk, New York, NY, USA) [58]. Descriptive statistics for categorical variables were summarized as frequencies and percentages, while each continuous variable was summarized in the form of the mean and standard deviation (SD). The prevalence of cyberbullying and suicidal behaviour among secondary school adolescents in Peninsular Malaysia were presented in the form of frequency and percentage. Simple and multiple binary logistic regression were used to examine the association between independent variables and suicidal behaviour as a binary outcome. The strength of association was estimated using the crude and adjusted odds ratio (COR and AOR) with a 95% confidence interval (CI). Independent variables associated with the outcome at the *p* < 0.25 level in the simple logistic regression were included in multiple logistic regression models using the forward stepwise selection method by likelihood-ratio test, the backward stepwise selection method by likelihood-ratio test, and manual selection methods. The significance level was based on *p* < 0.05.

## 3. Results

### 3.1. Sociodemographic and Other Characteristics of the Respondents

A total of 1290 respondents participated in the study, leading to an overall response rate of 89.6%. The number of girls was twice that of boys. The respondents were predominantly Malay (95.3%) and Muslim (95.7%). The mean age of the respondents was 14.48 (SD: 1.259). A majority of the respondents’ parents were married and living together (88.1%), and more than half of the respondents came from low-income households (52.2%). Most respondents perceived having a happy family (92.3%) and social support from family members (89.5%) and friends (89.1%). Among the respondents, 91.9% were social media users and regular Internet users (81.1%). Only 15.7% of the respondents had a history of abuse, and 0.6% of them had a history of substance abuse. On the other hand, 74.6% experienced academic pressure from their family, and 32.7% had shown symptoms of depression (Table 1).

### 3.2. Prevalence of Cyberbullying and Suicidal Behaviour among Secondary School Adolescents in Peninsular Malaysia

From Table 2, 13.7% of adolescents reported to be cyberbullying victims, and 3.8% were cyberbullying perpetrators. In addition, 2.4% of adolescents reported to be both cyberbullying victims and perpetrators. Table 2 also shows that 17.1% exhibited suicidal behaviour, in which having suicidal thoughts has the highest percentage (11.9%) compared to suicide plan and suicide attempt (Table 2).

### 3.3. Medium Used among Cyberbullying Victims and Perpetrators and Frequency of Cyberbullying Victimisation

The common medium used among cyberbullying victimization and perpetrators were instant messaging (62.7% and 57.1%, respectively) and text messaging (36.2% and 26.5%, respectively) (Table 3). Table 4 shows that the median score of cyberbullying victimization frequency was 2.0 (IQR: 6), whereby more than half (58.8%) of the students had a total score of less than or equal to 2 (Table 4).

### 3.4. Association between Cyberbullying and Suicidal Behaviour among Adolescents in Peninsular Malaysia

In simple logistic regression, cyberbullying victims and perpetrators were significantly associated with suicidal behaviour. Other variables also significantly associated with suicidal behaviour (*p* < 0.05) were age, gender, school locality, perceived social support from family members, perceived social support from friends, history of abuse, parents engaged in fight in front of children, frequency of cyberbullying victimization, and depression status. Multiple logistic regression analysis reported that cyberbullying victimization, age, gender, having parents who engaged in fight in front of their children, depression, history of abuse, and being perceived as having social support from family members and friends were significantly associated with suicidal behaviour (*p* < 0.05). Cyberbullying victims were reported to have a higher likelihood of suicidal behaviour than those who were not. Table 5 reported that girls had higher odds of suicidal behaviour than boys. Adolescents who had a history of abuse, whose parents engaged in fights in front of them, who had depression, and who perceived having no social support from family members and friends had a higher likelihood of suicidal behaviour than otherwise. Every one-year increase in age reduced the odds of suicidal behaviour by 19.0% (Table 5).

## 4. Discussion

From this study, we observed that 13.7% of adolescents were victims of cyberbullying and 3.8% of them reported being perpetrators of cyberbullying. Our findings differ from those of prior studies in Malaysia and other countries mainly because of the different questionnaires used, adolescent age groups, and the time when the studies were conducted. The prevalence of cyberbullying victimization among adolescents was 14.6% in South Korea [59], 7.2% in Australia [60], 8.8% in Spain [61] and 29.7% in Hong Kong, Taiwan, and Mainland China [62]. The prevalence of cyberbullying perpetrators among adolescents was 6.3% in South Korea [59], 3.5% in Australia [60], 3.1% in Spain [61], and 16.7% in Hong Kong, Taiwan, and Mainland China [62]. In Malaysia, [51] reported as high as 52.2% of adolescents in the state of Negeri Sembilan being involved in cyberbullying victimization while 31.6% of adolescents were victims of cyberbullying and 20.9% were perpetrators in the state of Penang [63]. The prevalence of adolescents who reported that they were both cyberbullying victims and perpetrators was consistent with previous research in the United States [64] and Sweden [65]. However, it was lower compared to other studies in South Korea [59], China [66] and Iran [67]. Cyberbullying prevalence varies across the states as some states, particularly the more urban ones, may have greater access to technology, thus leading to a higher prevalence of cyberbullying. Our study’s prevalence of cyberbullying victimisation and perpetrators fits within the vast range of previous prevalence estimates of 5–72% and 3.1–46.3%, respectively [3,51,59,60,61,62,63,68,69,70,71,72,73,74], which indicate a serious need to pay more attention to the cyberbullying problem. Our study was conducted during the COVID-19 pandemic and MCO when all schools in Malaysia were temporarily closed; hence, students used online learning to continue their home-based learning, which posed a high risk for cyberbullying due to the increase in Internet and social media usage [75,76,77]. The increased adolescent presence in cyberspaces is consistent with our finding, showing that 91.9% of the adolescents were social media users and regular Internet users, which might have contributed to the prevalence of cyberbullying among adolescents. In addition, the most common medium used by cyberbullying victims and perpetrators in our study were instant messaging and text messages, which were consistent with other studies [15,68,78,79]. 

Suicide prevalence estimates differ substantially between countries and studies [80]. According to our findings, 17.1% of adolescents reported suicidal behaviour with 11.9% having suicidal thoughts, 10.2% having suicide plans, and 8.4% who had attempted suicide. These findings were similar to those reported in Germany [81], Europe, Asia and the Western Pacific Region [82] and meta-analysis performed among adolescents between 1989 and 2018 [83]. Furthermore, suicide prevalence in our study was lower than that reported in Mozambique [36] and the Americas region [82,84]. However, the suicide prevalence in our study was higher than that reported in earlier Malaysian research (prevalence of suicidal thought = 10.0%, attempt = 7.3%, and plan = 6.9%) [52]. The differences in the study findings could be explained by variances in suicidal behaviour measurement as well as changes in the context of the time and study location. For example, Refs. [82,83] reported an incidence within a span of 365 days in the European and Western Pacific region while [84] reported the occurrence in their lifespan in the United States.

We discovered that adolescents who had been victims of cyberbullying were more prone to engage in suicidal behaviour than those who had not been victims of cyberbullying. This discovery was in line with previous research [7,8,9,15,23,85,86,87]. Cyberbullying victimisation has been shown to have an association with an increased risk of depression and anxiety symptoms, which may predispose to suicidal behaviour [7,8,86,87]. In our research, 32.7% of adolescents reported that they were depressed, and we also discovered a positive association between depression and suicidal behaviour. However, there was no significant association of suicidal behaviour with both cyberbullying perpetrators and the frequency of cyberbullying victimization in our study. Thus, our results were in contrast with other studies showing a positive association of adolescent suicidal behaviour with cyberbullying perpetrators and with the frequency of cyberbullying victimization [9,15,88]. This finding could be explained by the limited number of cyberbullying perpetrators identified in our study (3.8%).

Adolescents with depression were more likely than those without depression to engage in suicidal behaviour. This finding was consistent with other studies [25,33,34,85,89,90]. Suicide risk increased by 47–74% in people with mental illnesses, and 50–65% of suicide occurrences were associated with depression [90]. Academic pressure (academic-related stressor) was recognised as one of the top stressors among secondary school adolescents across various types of schools where they were pressured to maintain a strong academic performance in school [42,91,92]. More than half of the adolescents in our study reported that they were under academic stress. As a result, adolescents are under a great deal of stress, which in certain situations can lead to depression, making a person feel hopeless and helpless, and leading them to consider suicide as the only way to end their suffering [92]. Almost half of the adolescents who responded to our survey came from full-boarding schools, where students were expected to excel academically while being away from home, thus leading to high academic stress [93].

In our study, other factors were also reported to be significantly associated with suicidal behaviour included age, gender, having parents who engaged in fights in front of their children, history of abuse, and perceived social support from family members and friends. We revealed that increasing age protected adolescents from suicidal behaviour. This was substantiated by [24], who found that younger adolescents were at a higher risk of suicidal behaviour. Younger suicide victims were observed to have poor problem-solving skills [90]. Girls were shown to be more likely than boys to engage in suicide behaviour, which was consistent with findings from other studies [25,26,27,94,95]. Disparities in mental and behavioural difficulties may explain some of the differences in suicidal behaviour risk between boys and girls [96]. Adolescents whose parents fought in front of them were more likely to demonstrate suicidal behaviour than those whose parents did not. Having parents who fight in front of their children indicates a potentially unstable family situation, which is a risk factor for suicide behaviour in adolescents [38,39,40]. This result was confirmed by other reports showing a significant association between the family environment with suicidal behaviour among adolescents [38,39,40,97]. We also discovered that adolescents who perceived having no social support from family members or friends were more likely to engage in suicidal behaviour than others. Such a finding was consistent with other studies [24,34,82,94,98]. Adolescents are less likely to discuss their difficulties due to the lack of social support from family and peers, making them feel more distressed [98,99]. Teenagers were under a significant level of stress as a result of the COVID-19 pandemic, such as school cancellation and social isolation [100]. During the pandemic, the lack of social support was recognised as one of the risk factors for suicidality in youth [101,102]. The physical and social separation required to prevent COVID-19 from spreading further might have caused adolescents to feel a loss of support, thus increasing their risk of suicide. In addition, the lack of direct social interactions with peers has increased loneliness and social isolation, both of which can increase the likelihood of suicidal behaviour [102,103,104]. Our research also reported that adolescents who admitted that they had been abused were more likely to engage in suicidal behaviour than those who indicated otherwise. This finding is consistent with previous studies showing a significant association between a history of abuse and suicidal behaviour among adolescents [31,32,33,105,106,107]. Movement restriction caused by the pandemic lockdowns led to a surge in recorded incidences of child abuse, neglect, and exploitation [108,109]. Cases of child sexual assault also increased as a result of the simultaneous lockdown measures [110].

### 4.1. Strengths of the Study

There are a few strengths to be highlighted in this study. As we included adolescents from all zones, the sample in our study may represent Malay adolescents in Peninsular Malaysia. To our knowledge, this is the second local study looking at the association between cyberbullying and suicidal behaviour among school-going adolescents, and our sample size was larger than the first local study. The study was conducted in Malay, which is the local population’s native language, to ensure greater language accessibility and understanding. All study instruments had a Malay version that has been validated with good reliability and validity results. 

The use of a self-administered questionnaire using web-based mode in our study was cost effective. In addition, the survey could be administered concurrently within a larger scope, meaning less time spent on administration [111]. Using an online and self-administered survey also ensures high willingness of respondents to disclose sensitive information due to the assurance of anonymity [112,113,114], which is vital because our study touches on sensitive issues such as suicidal behaviour, history of abuse, and interpersonal issues. Furthermore, since the data collection took 5 months which occurred within the period of the movement control order (MCO) due to the COVID-19 pandemic, there was no variation in MCO restriction policies from May to September 2021. Therefore, the variation in responses could be minimized. 

We were also able to quantify the level of association between the explanatory variable (cyberbullying) and the outcome variable (suicidal behaviour) using multiple logistic regression while controlling for a number of confounders [115].

### 4.2. Limitations

Several limitations were encountered in this study and should be considered in the interpretation of the findings. A cross-sectional design does not establish a temporal link between the exposure (cyberbullying) and the outcome (suicidal behaviour) [116]. The majority of respondents in our survey were Malays as they make up the largest ethnic group in the Malaysian population. This could limit the applicability of the findings to other ethnic groups. Adolescents with disabilities, particularly those with intellectual disabilities, were not included in our study. Suicidal behaviour among adolescents may be influenced by those having a disability [117]. Therefore, future research involving a larger number of other ethnic groups (i.e., increasing ethnic diversity) and adolescents with disability is recommended. In addition, qualitative research on identifying other suicide factors such as cultural factors or socio-cultural changes in the youth environment due to the availability of the Internet and other communication tools such as smartphones or a reaction to the threats and restrictions related to the pandemic is required. Furthermore, the usage of the word ‘suicide’ in the suicide questions might have drawn students’ attention and provoked affirmative answers. Nonetheless, these questions were routinely used to assess suicidal behavior among adolescents in the national local survey in Malaysia [52]. A history of chronic illness and mental illness were self-reported; thus, it could be under- or over-reported. As data collection was conducted online and self-reported, we were not able to query or verify the information submitted. Due to this reason, we were also not able to collect data from those living in rural areas with limited internet access, thus preventing them from participating in our study. One possible strategy to improve the validity and reliability of the self-report data is to follow up with the respondents using qualitative methods with the involvement of mental health experts being recommend in future research.

## 5. Conclusions

In conclusion, this study contributed to the knowledge on cyberbullying and suicidal behaviour among adolescents in Malaysia, particularly during the COVID-19 pandemic. It revealed an astonishing number pertaining to the prevalence of cyberbullying and suicidal behaviour among adolescents in Peninsular Malaysia. Cyberbullying victims were found to be a risk factor for suicide. Other risk factors included young age, history of abuse, being a girl, living in an unstable family, depression, and the perception of no social support from family and friends. Our findings warrant the need to strengthen public awareness on cyberbullying and its impact on mental health, as well as more effective strategies focusing on the early identification and intervention of adolescents with a higher risk for suicide.

## Figures and Tables

**Table 1:** Sociodemographic and other characteristics of the respondents (n = 1290).

Variables	n	%
**Age (total sample) ^a^**	14.48	1.259
**Gender**		
Female	905	70.2
Male	385	29.8
**Type of school**		
National Secondary School (Daily)	336	26
Full Boarding School (SBP)	623	48.3
Religions Secondary Schools (SMKA) and Government Aid Religious School (SABK)	331	25.7
**School locality**		
Rural	679	52.6
Urban	611	47.4
**Form**		
Form 1	369	28.6
Form 2	429	33.3
Form 4	492	38.1
**Race**		
Malay	1229	95.3
Chinese	50	3.9
Others ^b^	11	0.8
**Religion**		
Islam	1234	95.7
Buddha	44	3.4
Others ^c^	12	0.9
**Parental marital status**		
Married and living together	1136	88.1
Married but not living together (Working elsewhere)	28	2.2
Divorced	57	4.4
Widow (Father or mother who has passed away)	46	3.6
Separated (Parents not living together)	11	0.9
Do not know	12	0.9
**Household income**		
Less than RM 4850 (B40)	673	52.2
RM 4850—RM 10,959 (M40)	458	35.5
More than RM 10,959 (T20)	159	12.3
**Perceived social support from family members**		
Yes	1155	89.5
No	135	10.5
**Perceived social support from friends**		
Yes	1150	89.1
No	140	10.9
**Having social media account**		
Yes	1185	91.9
No	105	8.1
**Duration of internet use**		
1 h per day	28	2.2
2–3 h per day	216	16.7
More than 3 h per day	1046	81.1
**History of abuse (physical/mental/sexual)**		
Yes	202	15.7
No	1088	84.3
**Perceived having academic pressure**		
Yes	962	74.6
No	328	25.4
**Perceived having happy family**		
Yes	1191	92.3
No	99	7.7
**Parents engage in fighting in front of adolescents**		
Yes	157	12.2
No	1133	87.8
**History of chronic illness**		
Yes	56	4.3
No	1234	95.7
**History of mental illness**		
Yes	30	2.3
No	1260	97.7
**History of substance abuse**		
Yes	8	0.6
No	1282	99.4
**Depression status**		
Yes	422	32.7
No	868	67.3

^a^ Age presented in mean and standard deviation; ^b^ Other races (Indian, Bumiputera Sabah, Bumiputera Sarawak, Kelabit); ^c^ Other religion (Christian, Hindu, none). Note: due to rounding, percentages do not always add up to 100.

**Table 2:** Prevalence of cyberbullying and suicidal behaviour among adolescents in secondary schools in Peninsular Malaysia (n = 1290).

Variables	n (%)
**Cyberbullying victimization**	
Yes	177 (13.7)
No	1113 (86.3)
**Cyberbullying perpetrator**	
Yes	49 (3.8)
No	1241 (96.2)
**Cyberbullying victimization perpetrators**	
Yes	31 (2.4)
No	1259 (97.6)
**Suicidal behaviour**	
Yes	221 (17.1)
No	1069 (82.9)
**Suicidal thought**	
Yes	154 (11.9)
No	1136 (88.1)
**Suicide plan**	
Yes	132 (10.2)
No	1158 (89.8)
**Suicide attempt**	
Yes	108 (8.4)
No	1182 (91.6)

Note Due to rounding, percentages do not always add up to 100.

**Table 3:** Medium used for cyberbullying victimization and perpetrators.

Medium Used	Cyberbullying Victimization (n = 177)	Cyberbullying Perpetrator (n = 49)
	n (%)	n (%)
**Email**		
Yes	3 (1.7)	2 (4.1)
No	174 (98.3)	47 (95.9)
**Text messages**		
Yes	64 (36.2)	13 (26.5)
No	113 (63.8)	36 (73.5)
**Picture messages**		
Yes	38 (21.5)	6 (12.2)
No	139 (78.5)	43 (87.8)
**Instant messaging**		
Yes	111 (62.7)	28 (57.1)
No	66 (37.3)	21 (42.9)
**Developed a mean website or message board for you**		
Yes	26 (14.7)	4 (8.2)
No	151 (85.3)	45 (91.8)
**Online video clips of you**		
Yes	17 (9.6)	2 (4.1)
No	160 (90.4)	47 (95.9)
**Social networking site (such as Facebook)**		
Yes	8 (4.5)	3 (6.1)
No	169 (95.5)	46 (93.9)
**Chatroom**		
Yes	37 (20.9)	8 (16.3)
No	140 (79.1)	41 (83.7)
**Virtual world (such as Second Life or the Sims)**		
Yes	25 (14.1)	5 (10.2)
No	152 (85.9)	44 (89.8)

Note Due to rounding, percentages do not always add up to 100.

**Table 4:** Frequency of cyberbullying victimization (n = 1290).

Variable	Median (IQR)	n (%)
Total score of CBS scale (item 3–16)	2.0 (6.0)	
Score ≤ 2		758 (58.8)
Score > 2		532 (41.2)

IQR = interquartile range. Note: due to rounding, percentages do not always add up to 100.

**Table 5:** The association between cyberbullying and suicidal behaviour among secondary school adolescents in Peninsular Malaysia adjusted for other confounders analysed by simple and multiple logistic regression.

Variables	Suicidal Behaviour, n = 221	No Suicidal Behaviour, n = 1069	Crude Odds Ratio (95% CI)	*p*-Value ^b^	Adjusted Odds Ratio (95% CI)	*p*-Value ^c^
	n (%)	n (%)				
**Cyberbullying victimization**						
No	137 (62.0)	976 (91.3)	1		1	
Yes	84 (38.0)	93 (8.7)	6.44 (4.56, 9.09)	<0.001	2.35 (1.50, 3.69)	<0.001
**Cyberbullying perpetrator**						
No	205 (92.8)	1036 (96.9)	1			
Yes	16 (7.2)	33 (3.1)	2.45 (1.32, 4.54)	0.004		
**Frequency of cyberbullying victimization**						
≤2 (Less frequent)	61 (27.6)	697 (65.2)	1			
>2 (More frequent)	160 (72.4)	372 (34.8)	4.92 (3.57, 6.77)	<0.001		
**Gender**						
Male	25 (11.3)	360 (33.7)	1		1	
Female	196 (88.7)	709 (66.3)	3.98 (2.58, 6.15)	<0.001	3.64 (2.11, 6.25)	<0.001
**Age ^a^**	14.3 (1.21)	14.5 (1.27)	0.86 (0.76, 0.97)	0.011	0.81 (0.69, 0.94)	0.005
**School locality**						
Rural	102 (46.2)	577 (54.0)	1			
Urban	119 (53.8)	492 (46.0)	1.37 (1.02, 1.83)	0.034		
**Type of school**						
National Secondary School (Daily)	56 (25.3)	280 (26.2)	1	0.176		
Religions Secondary Schools (SMKA) and Government Aid Religious School (SABK)	47 (21.3)	284 (26.6)	0.83 (0.54, 1.26)	0.378		
Full Boarding School (SBP)	118 (53.4)	505 (47.2)	1.17 (0.82, 1.66)	0.384		
**Parental marital status**						
Stable parental marital status (Married and living together)	192 (86.9)	944 (88.3)	1			
Unstable parental marital status (Married but not living together (Working elsewhere)/Divorced/Widow (Father or mother who has passed away)/Separated (Parents not living together)/Do not know)	29 (13.1)	125 (11.7)	1.14 (0.74, 1.76)	0.551		
**Household income**						
Stable (RM 4850—RM 10,959 [M40]/More than RM 10,959 [T20])	108 (48.9)	509 (47.6)	1			
Poor (Less than RM 4850 [B40])	113 (51.1)	560 (52.4)	0.95 (0.71, 1.27)	0.734		
**Perceived social support from family members**						
Yes	151 (68.3)	1004 (93.9)	1		1	
No	70 (31.7)	65 (6.1)	7.16 (4.90, 10.46)	<0.001	2.49 (1.52, 4.09)	<0.001
**Perceived social support from friends**						
Yes	166 (75.1)	984 (92.0)	1		1	
No	55 (24.9)	85 (8.0)	3.84 (2.63, 5.59)	<0.001	1.96 (1.21, 3.19)	0.006
**Having social media account**						
No	12 (5.4)	93 (8.7)	1			
Yes	209 (94.6)	976 (91.3)	1.66 (0.89, 3.08)	0.109		
**History of abuse (physical/mental/sexual)**						
No	132 (59.7)	956 (89.4)	1		1	
Yes	89 (40.3)	113 (10.6)	5.70 (4.09, 7.95)	<0.001	2.28 (1.47, 3.54)	<0.001
**Perceived having academic pressure**						
No	45 (20.4)	283 (26.5)	1			
Yes	176 (79.6)	786 (73.5)	1.41 (0.99, 2.01)	0.058		
**Parents engage in fighting in front of children**						
No	159 (71.9)	974 (91.1)	1		1	
Yes	62 (28.1)	95 (8.9)	4.00 (2.79, 5.74)	<0.001	2.19 (1.38, 3.49)	0.001
**Depression status**						
No	40 (18.1)	828 (77.5)	1		1	
Yes	181 (81.9)	241 (22.5)	15.55 (10.73,22.54)	<0.001	7.54 (5.04, 11.28)	<0.001

^a^ Age presented in mean and standard deviation. ^b^ Simple Logistic Regression. ^c^ Multiple Logistic Regression. Stepwise Forward, Backward LR and manual selection method were applied. There is no multicollinearity and interaction. Classification table classified correctly 86.7%. Area Under ROC curve was 88.8%. Note: due to rounding, percentages do not always add up to 100.

## Data Availability

The data presented in this study are available from the corresponding author on reasonable request. The data are not publicly available due to ethical concerns.
